# Eps15: a multifunctional adaptor protein regulating intracellular trafficking

**DOI:** 10.1186/1478-811X-7-24

**Published:** 2009-10-08

**Authors:** Paul MP van Bergen en Henegouwen

**Affiliations:** 1 Cellular Architecture & Dynamics, Institute of Biomembranes, Utrecht University, The Netherlands

## Abstract

Over expression of receptor tyrosine kinases is responsible for the development of a wide variety of malignancies. Termination of growth factor signaling is primarily determined by the down regulation of active growth factor/receptor complexes. In recent years, considerable insight has been gained in the endocytosis and degradation of growth factor receptors. A crucial player in this process is the EGFR Protein tyrosine kinase Substrate #15, or Eps15. This protein functions as a scaffolding adaptor protein and is involved both in secretion and endocytosis. Eps15 has been shown to bind to AP-1 and AP-2 complexes, to bind to inositol lipids and to several other proteins involved in the regulation of intracellular trafficking. In addition, Eps15 has been detected in the nucleus of mammalian cells. Activation of growth factor receptors induces tyrosine phosphorylation and mono-ubiquitination of Eps15. The role of these post translational modifications of Eps15 is still a mystery. It is proposed that Eps15 and its family members Eps15R and Eps15b are involved in the regulation of membrane morphology, which is required for intracellular vesicle formation and trafficking.

## Introduction

Receptor tyrosine kinases (RTK) are a large family of signaling proteins involved in a large number of human diseases. They all have a similar composition: an extracellular domain that binds to a growth factor, a trans-membrane domain, an intracellular tyrosine kinase domain and a stretch of tyrosine residues that serves as substrates for the kinase. Binding of the growth factor results in kinase activation and consequently in the trans-phosphorylation of the receptor, as well as of various effector molecules resulting in the stimulation of a large number of signaling cascades. One of the most studied RTKs is the epidermal growth factor (EGF) receptor (EGFR or ErbB1), which belongs to a family of four related receptor tyrosine kinases (ErbB1-4 or Her1-4)). EGFR and its family members are strongly implicated in the development and progression of different human tumors, including breast-, lung-, prostate-, colorectal-, head and neck- and brain tumors [1]. These cancers are often correlated with receptor over-expression and/or mutations in the receptor tyrosine kinase, frequently associated with poor prognosis for patients [2].

Attenuation of RTK signaling is governed by several mechanisms. At the receptor level, tyrosine phosphatases reduce the number of phosphorylated tyrosine residues. At the cellular level, inhibition of signaling is accomplished by receptor desensitization or down regulation. This process involves the internalization of active ligand/receptor complexes and subsequent trafficking to lysosomes, where receptors are degraded [3]. Ubiquitination of the RTKs is considered as an important step both in the recruitment of receptors into coated pits and in the sorting process in the early endosome [4,5]. Aberrant expression of regulators of endocytosis and consequently of receptor down regulation is strongly related to the development of many different cancers [6]. For instance, abolishment of the ubiquitination of EGFR by mutations in the involved E3 ligase, c-Cbl, has been found to result in oncogenic transformation [7]. The recruitment of active, ubiquitinated receptors into coated pits is an example of a process in which many different proteins with multiple protein:protein interactions are involved. One of the major players in this process is the scaffolding protein Eps15.

## A dual function for Eps15

**Figure 1:**
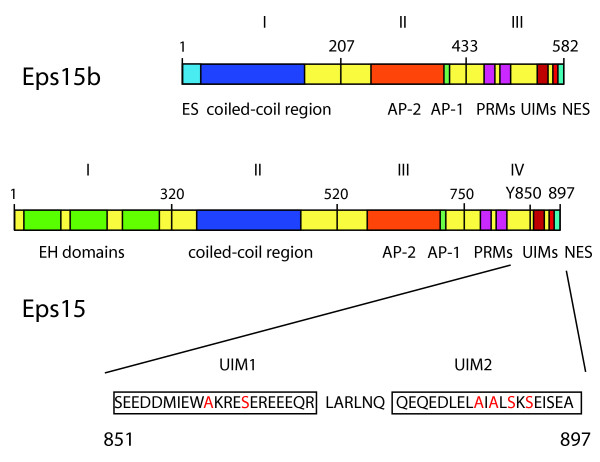
**Domain structure of the Eps15 proteins**. Different functional regions are indicated, green: Eps15 Homology or EH domains; light bleu: specific Eps15b region; dark blue: coiled-coil region; orange: binding site to α-adaptin; light green: binding site for γ-adaptin; purple: proline rich motifs (prm); red: ubiquitin interacting motifs (UIM) and blue: nuclear export signal (NES). Y850 tyrosine phosphorylation site in Eps15. Although Eps15b has multiple DPF motif it binds poorly to α-adaptin *in *vivo [29]. The sequences of the two UIMs are shown. Note the double serine and alanine motifs (in red) in the UIM2.

The EGFR Protein tyrosine kinase Substrate # 15 (Eps15) was originally identified in 1993 in a pool of proteins that became phosphorylated after stimulation of cells with EGF [8]. In the same period, Eps15 was identified as a binding partner of α-adaptin, a component of the AP-2 complex and part of clathrin-coated pits and vesicles [9]. A protein related to Eps15, Eps15R, was indentified as a binding partner of the oncogenic variant of the adaptor protein Crk (v-Crk) [10]. The v-Crk SH3 domain was found to bind to a proline rich sequence present in the C-terminal part of the protein (Fig. 1). The amino acid sequence of Eps15R is 41% identical and 61% similar to that of Eps15 [10]. Localization experiments using confocal microscopy showed co-localization of Eps15 with α-adaptin and clathrin, but not with rab4 and rab5, indicative for a localization in coated pits [11]. Similar results were obtained for Eps15R [12]. Interestingly, electron microscopy only revealed the presence of Eps15 at the rim of the coated pit [13].

The localization of Eps15 in coated pits strongly suggested the implication of Eps15 in the internalization of the EGFR. Different experimental approaches have now confirmed this role. Microinjection of antibodies against Eps15 were shown to inhibit the internalization of EGFR and of the transferrin receptor [14]. More recently, knockdown of both Eps15 and Eps15R showed a 40% inhibition of transferrin and EGF uptake indicating an important role for Eps15 and Eps15R in clathrin-mediated endocytosis [15]. Silencing of spartin, a binding partner of Eps15, has been shown to result in aberrant trafficking of the EGFR, which may underlie the pathogenesis of Troyer syndrome [16]. Troyer syndrome is a hereditary spastic paraplegia characterized by progressive spasticity and muscle weakness in the lower limbs. Similarly, binding of Eps15 with parkin has been shown to affect EGFR trafficking and signaling, which may be related to the development of Parkinson's disease [17].

In addition to the presence and role of Eps15 in the endocytic system, Eps15 and especially Eps15R have also been found in the nucleus [18]. Eps15, but not Eps15R contains a leucine-rich, nuclear export signal (NES) at its very C-terminus that keeps it out of the nucleus [19]. Import of Eps15R into the nucleus is independent of a nuclear localization signal (NLS) and requires binding to proteins like Hrb (HIV-1 Rev-binding protein) or another endocytic protein Epsin1 [20]. Regulation of the nuclear-cytoplasmic shuttling of Eps15 is not clear. Once in the nucleus, Eps15 has been shown to act as positive modulator of transcription in a GAL4-based transactivation assay, suggesting a direct or indirect role for Eps15 in transcriptional regulation [18]. Moreover, other endocytic proteins were also found in the nucleus, including intersectin, Epsin1, CALM, HIP1, Dab1/2 and β-arrestins. The Eps15 binding partner intersectin was shown to activate the Elk-1 transcription factor [21]. These observations suggest a dual function for endocytic proteins including the two Eps15 homologues: in membrane sorting during endocytosis and in regulating gene expression in the nucleus. Their exact role in the nucleus is, however, far from clear [22].

The Eps15 gene is located at chromosome 1 at chromosomal locus p31-p32, which displays a high rate of random chromosomal abnormalities such as deletions in oligodendroglioma and neuroblastoma, and translocations in myeloid and lymphoblastic leukemia [23]. Fusion of Eps15 to transcription factors has been found in certain forms of acute myeloid leukemia (AML), where Eps15 is fused to the mixed lineage leukemia gene (MLL) [24-26]. The MLL gene, which is also referred to as HRX, ALL1 or HTRX, encodes a transcription factor regulating the expression of Hox genes. These downstream targets of MLL encode transcription factors with critical roles in both embryonic and hematopoietic differentiation [27]. Fusion of the MLL protein with Eps15 resulted in dimerization of the fusion protein, which was found to activate this transcription factor [27]. The MLL-Eps15 fusion protein, also indicated as HRX-ALL-Eps15 fusion protein, was found exclusively in the nucleus, while the wild type MLL protein localized to both the cytoplasm and nucleus [28]. This suggests that the fusion to Eps15 induces both the activation and translocation of the protein to the nucleus, which may be responsible for the oncogenic potential of this fusion protein and the development of leukemia.

In summary, Eps15 is functioning in the cytoplasm, where it is involved in in the regulation of intracellular trafficking. In addition, its presence in the nucleus suggests a role for Eps15 in transcriptional regulation. To understand the working mechanism of Eps15 in its diverse physiological functions, a detailed analysis of its structure and molecular binding partners is required.

## Modular structure of the Eps15 proteins

Eps15 appears to be a member of a small group of proteins: Eps15, Eps15R and Eps15b. Both Eps15 and Eps15R are organized into four different domains (fig. 1). Eps15b lacks domain I, but contains a unique N-terminal stretch of 32 amino acids (fig. 1) [29]. Domain I contains three different Eps15 homology or EH domains, which were originally described as protein:protein interaction modules [30]. The EH domain is present in a great variety of endocytic proteins such as intersectin, γ-synergen, Reps1 and EH domain containing proteins (EHDs) ([31-33]. Structural analysis by NMR has shown that the EH domains consist of two anti-parallel oriented EF hands [34,35]. The EF hand is a helix-turn-helix motif with Ca^2+ ^binding properties, consisting of two α-helices linked by a short β-strand. The EH domains of Eps15 have been found to bind Ca^2+ ^constitutively with high affinity. Phage display analysis has identified the protein motif this domain binds to, being composed of the three amino acid motif asparagine, proline and Phenylalanine (NPF) for the first two domains [36]. The third EH domain displays a different specificity: FW containing motifs preferentially bind to this domain. Proteins containing EH domains are predominantly involved in intracellular trafficking, suggesting a general role for EH domains in intracellular transport. GST-pull downs and yeast-two hybrid searches have yielded several different binding partners of the EH domains of Eps15, including the endocytic proteins Epsin1, STAM (EAST), Stonin2, Numb, synaptojanin and others (see table 1). The EH1 and EH4 domain of EHD1 and the EH2 domain of Eps15 were found to interact with phosphatidyl-inositol lipids [37]. NMR studies indicated positively charged lysine residues as critical for the phospho-inositide binding, and these residues are also present at homologous positions in the Eps15-EH2 domain [37]. EHD proteins can induce membrane curvature *in vitro *and are also localized to the tubular structures of endosomal membranes [38,39]. Although the EHD1 is required for recycling of transferrin receptors, their function in the induction of membrane curvature *in vivo *remains to be seen [39].

**Table 1:** Eps15 binding partners

**Binding partner**	**Interacting domain**	**Function**	**Reference**
**α-adaptin**	DPF-motif	Endocytosis	[64]

**γ-adaptin**	Domain III	secretion	[44,45]

**Crk**	PRMs	Unknown	[10]

**Eps15/Eps15R**	EH domain	Unknown	[46]

**Epsin**	EH domain	Endocytosis/nucleus	[51]

**Grb2**	PRMs	Endocytosis	[10,42]

**Hrb**	EH domain	Nucleus/endocytosis	[77,78]

**Hrs**	n.d.	Sorting	[74]

**Intersectin**	Coiled-coil domain	Endocytosis	[41]

**Numb**	EH domain	Endocytosis	[36]

**Parkin**	UIM domains	Mono-ubiquitination	[17]

**POB1**	n.d.	Endocytosis	[79]

**Phocein**	n.d.	Trafficking	[80]

**Spartin**	n.d.	Endocytosis	[81]

**STAM (EAST)**	n.d.	Sorting	[74]

**Stonin2**	EH domain	Endocytosis	[53]

**Synaptojanin**	EH domain	Endocytosis	[82]

**Ubiquilin/PLIC**	UIM1	Aggresome formation	[49]

**Ubiquitin**	UIM2	Ubiquitination/unknown	[59,60]

n.d.: not determined

The second domain of the Eps15, Eps15R and Eps15b consists of a coiled-coil region, which has the capacity to dimerize (fig. 1). As a result, Eps15 has been found as parallel and anti-parallel dimers and tetramers [40]. Moreover, this region has been shown to interact with the endocytic protein intersectin [41]. Recently, co-precipitation of Eps15 with the EGFR and c-Met receptor was shown to depend on the coil-coiled region [42]. Whether this interaction is direct or mediated by other proteins is not known.

The third domain is characterized by the presence of several DPF motifs. These motifs play a role in the association with the appendage or ear of α-adaptin, a component of the AP-2 complex [43]. C-terminal from the AP-2 binding site is a small motif present, which is responsible for a direct binding to γ-adaptin, a component of the AP-1 complex ([44,45]. Remarkably, the DPF motifs have also been shown to function as ligands for the EH domains [46]. This enables the formation of larger networks of Eps15, consisting of Eps15 dimers that are connected to each other via DPF/EH interactions. The question arises whether those complexes exist *in vivo *and whether their interactions with inositol lipids would facilitate self-association of Eps15. Indications for the existence of stable Eps15 structures were obtained using isolated membrane sheets. Staining of such ventral membranes with anti-Eps15 antibodies showed the typical coated pit pattern and colocalization with clathrin and α-adaptin [11]. After extraction of both clathrin and α-adaptin from the membrane the typical Eps15 distribution was still present, suggesting that Eps15 itself could form a stable structure.

The fourth domain of Eps15 can be indicated as the regulatory domain, as it contains at position 850 the tyrosine residue that becomes phosphorylated upon stimulation of the cell with EGF and HGF (Hepatocyte Growth Factor) [42,47]. Endocytosis of EGFR could be specifically inhibited by over-expression of an Eps15 mutant lacking this site [47]. Domain IV also contains two proline rich domains (prm), which were found capable of binding to the adaptor proteins Crk and Grb2 [10]; the latter was also shown to co-immunoprecipitate with the c-Met receptor [42]. The nuclear export signal is located at the very C-terminus of Eps15 and Eps15b but is absent in Eps15R [19]. In this part of Eps15, two ubiquitin interacting motifs (UIM) are present of which the UIM2 sequence overlaps with the NES [48]. These UIMs indeed bind to (poly)ubiquitinated proteins, but binding to proteins containing a Ubiquitin like domain (Ubl) such as ubiquilin-1 or PLIC has also been observed [49]. Interestingly, the UIM1 appears to have higher affinity for this Ubl domain than for ubiquitin, while the reverse was observed for the UIM2. Among the highly conserved residues in the UIM domains are the serine (S) and alanine (A) residues (fig. 1). UIM domains adopt an α-helical structure and the S and A residues are located at one site of the α-helix. The Eps15-UIM2 has two serine and alanine residues that are located opposite of each other in the α-helix. This has lead to the hypothesis that the UIM2 can actually bind to two ubiquitin moieties at the same time [50]. This would explain the higher affinity of this UIM for poly-ubiquitinated proteins as compared to that of UIM1.

## Eps15 binding partners

Essential for an understanding of the function of Eps15 is the determination of the protein binding partners of this molecule. An overview of all known binding partners of Eps15 is given in Table 1. One of the first Eps15 binding partners that has been discovered was Epsin1, which binds directly with its NPF motif to the first two EH domains [51] of Eps15. Besides the NPF motif, the Epsin family of proteins contains a lipid-binding domain, the ENTH domain. Elegant electron microscopy studies have shown that binding of Epsin to the membrane induces a bending of the membrane, an essential step in the invagination process of the membrane required for vesicle formation [52].

Searches for Eps15 binding partners using yeast-two-hybrid screens have yielded many interesting new targets, for instance Numb and Stonin2 (Table 1). Both proteins are involved in the process of receptor internalization: Numb in the internalization of the Notch receptor while Stonin-2 is involved in the internalization of the receptor for transferrin, EGF and LDL [53-55]. Several other endocytic proteins have been found to bind Eps15, including Hrs, STAM, synaptojanin, POB1, α- and γ-adaptin and intersectin, all pointing towards the essential role of Eps15 in secretion and internalization processes (Table 1).

The two UIMs in Eps15 were found to bind to at least two ubiquitin E3-ligases, Nedd4 and Parkin (see below). Interestingly, the first UIM of Eps15 has also been found to bind to the Ubl-domain of ubiquilin or PLIC. Colocalization experiments demonstrated the presence of Eps15 and ubiquilin in intracellular aggregates [49]. Subsequent gene silencing experiments of Eps15 demonstrated that the association of Eps15 with ubiquilin is involved in the formation of aggresomes and possibly also in the removal of these aggregates [56]. Involvement of ubiquilin in autophagy was recently confirmed by studies from N'Diaye and coworkers who demonstrated this role of ubiquilin in autophagy-dependent cell survival during nutrient starvation [57]. This suggests the possibility that Eps15, as an endocytic protein, might also be involved in autophagy, the cellular process that is involved in the degradation of large protein aggregates. However, more research is required to better understand the role of Eps15 in the removal of protein aggregates.

## Post translational modifications of Eps15: phosphorylation and ubiquitination

Eps15 was initially found as a tyrosine kinase substrate of the EGFR. Further research indicated that the tyrosine at position 850 acts at the target tyrosine residue [47]. Over expression of an Eps15 mutant lacking this site could specifically block the endocytosis of the EGFR, but not that of the transferrin receptor [47]. The involvement of a phosphorylated tyrosine residue in the functioning of Eps15 suggest the binding to a phosphotyrosine binding protein, which may recruit Eps15 either to the receptor or to the endocytic machinery. However, such proteins that may contain an SH2 or PTB domain have not been identified as yet.

Stimulation of the cell with EGF results in a remarkable shift in the molecular weight of Eps15 and Eps15R [8]. This modification was found to be the result of the conjugation of one ubiquitin moiety: Eps15 mono-ubiquitination [58]. Detailed mapping of the structural elements present in Eps15 that are required for ubiquitination indicated an essential role for the UIM motifs [59,60]. Subsequently, the hunt was on for the Eps15 E3-ligase(s) that could both bind to the Eps15-UIMs and ubiquitinate the protein. Two E3 ligases have so far been identified: Nedd4 and parkin, both acting via different mechanisms [17,61].

**Figure 2:**
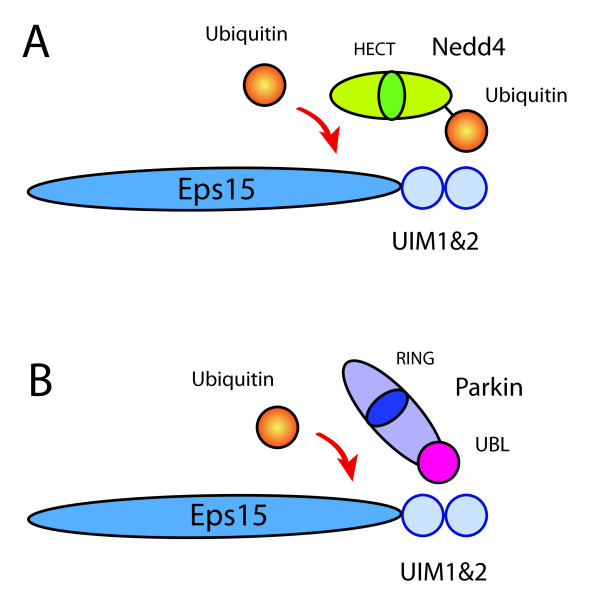
**Different mechanisms for Eps15 ubiquitination**. The E3 ligases Nedd4 and parkin ubiquitinate Eps15 by different mechanisms: Nedd4 needs to be ubiquitinated before binding to the Eps15-UIM can occur. Parkin contains a UBL domain which binds constitutively to the UIMs of Eps15. HECT and RING domains are indicated in their respective E3 ligases.

In yeast, the E3 ligase Rsp5 has been implicated in numerous cellular functions including protein degradation and endocytosis [62]. Nedd4 can be considered as the human orthologue of Rsp5 and this ligase was previously found on endocytic vesicles [62]. Dr. Polo and colleagues investigated whether Nedd4 could act as a possible E3-ligase for Eps15 [59]. Over-expression of Nedd4 resulted in an increased ubiquitination of Eps15. In addition, purified Nedd4 could ubiquitinate Eps15 in an *in vitro *ubiquitination assay [61]. A pre-incubation of Nedd4 in the *in vitro *ubiquitination mixture clearly enhanced the mono-ubiquitination of Eps15. These elegant studies demonstrated that Nedd4 is first self-ubiquitinated, which allows it to bind the second UIM2 of Eps15. Subsequently, Eps15 becomes ubiquitinated by the action of Nedd4 (fig. 2). In addition, the authors suggested that upon conjugation of one single ubiquitin moiety to Eps15, this ubiquitin moiety may bind to the UIM2 on the same molecule, resulting in the release of the Nedd4 and prevention of poly-ubiquitination of Eps15 [61].

Following the finding that UIM motifs can also interact with ubiquitin like domains (Ubl), E3 ligases were screened for possessing an Ubl domain. This yielded Parkin, an E3 ligase related to the development of Parkinson's disease [17]. It was shown that the Ubl domain of parkin binds to the UIM motifs of Eps15, which interaction is required for the mono-ubiquitination to occur (fig. 2). In this way, Eps15 was ubiquitinated in an *in vitro *ubiquitination assay, and over-expression of parkin resulted in an increase in mono-ubiquitination of Eps15. Possibly, a similar competition between the conjugated ubiquitin and parkin for binding to the UIM2 may be responsible for the prevention of poly-ubiquitination of Eps15.

Although both E3 ligases Nedd4 and parkin are clearly ubiquitinating Eps15, many questions remain unanswered. For instance, Nedd4 and parkin are both members of the two different classes of E3 ligases: Nedd4 contains a C-terminal catalytic HECT domain, short for homologous to the carboxyl terminus of the E3 ligase E6-AP, while parkin contains a RING domain, short for really interesting new gene. It is currently unclear how EGF-mediated signaling would stimulate this parkin/Eps15 association and consequently stimulate Eps15 mono-ubiquitination. Woelk and coworkers have shown that also other HECT domain E3 ligases are ubiquitinated, and the question arises whether all these ubiquitinated ligases can also bind to Eps15, and consequently mono-ubiquitinate Eps15 [61]. Probably, other additional factors may be involved to guarantee specificity in the binding of Eps15 with these HECT domain E3 ligases.

The most intriguing question remains the function of Eps15 ubiquitination. As over expression of parkin was found to enhance Eps15 mono-ubiquitination considerably, this offered the possibility to study the effect of Eps15 mono-ubiquitination on EGFR internalization. Parkin over expression clearly inhibited the internalization and degradation of the EGFR, while silencing of parkin clearly stimulated endocytosis [17]. Consequently, EGF-mediated signaling towards PKB/Akt was stimulated under parkin over expression conditions. These results suggested that the mono-ubiquitination of Eps15 may result in a functional inhibition possibly by the formation of an intra-molecular binding whereby the attached ubiquitin binds to the UIM2 of the same molecule. This intramolecular binding may prevent binding to α-adaptin and, as a result inhibit recruitment of Eps15 to the coated pit, which may cause the inhibition of EGFR endocytosis. A similar mechanism has been demonstrated for the Cbl-binding protein Sts2 using Förster Resonance Energy Transfer (FRET) of fluorescent proteins fused to the N- and C-terminus of Sts2 [63]. However, intramolecular binding of attached ubiquitin to the UIM2 of Eps15 has not been demonstrated as yet. Determination of the ubiquitination sites in Eps15 would be required for this kind of experiments and we are currently mapping these ubiquitination sites.

## Eps15 and intracellular trafficking

Initial observations indicated the direct binding of Eps15 to α-adaptin and the co-localization of Eps15 with α-adaptin and the heavy chain of clathrin in coated pits [9,11,64]. Involvement of Eps15 in the internalization of growth factor receptors has thus far been demonstrated for the EGFR and for the c-Met receptor [42,47]. In both cases, stimulation of the cell with ligand results in Eps15 phosphorylation and ubiquitination. Eps15 then associates with particular receptor in the plasma membrane [42,65]. The colocalization with the EGFR could be prevented by over-expression of the Eps15-UIM motifs while the induced association with the c-Met receptor was prevented by over expression of the coil-coiled domain of Eps15 (domain II) [42,66]. Thus, binding of Eps15 to the ubiquitinated EGFR probably occurs via interactions of the Eps15-UIMs with the ubiquitin moieties present on the intracellular domain of EGFR. A similar mechanism has been reported for the internalization of the junction protein occludin that becomes ubiquitinated and internalized after stimulation of the cell with VEGF [67]. The interaction of Eps15 with the c-Met receptor is more complex. Besides a possible direct interaction of the coil-coiled domain with the cMet receptor, the adaptor protein Grb2 may contribute to this association: Grb2 could possibly bind the c-Met receptor via its SH2 domain (recognizing the phosphorylated receptor), while its SH3 domain interacts with the prm in Eps15 (Fig. 1)[42].

**Figure 3:**
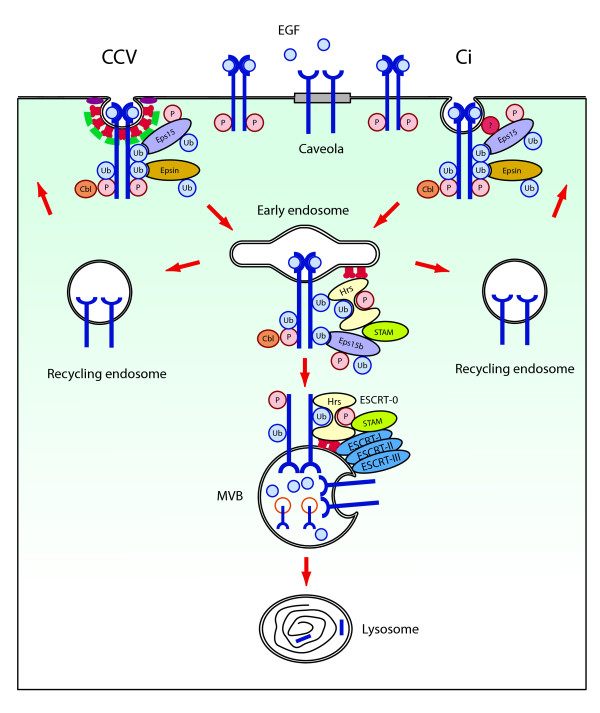
**Possible role of the Eps15 proteins in intracellular trafficking of EGFR**. EGFRs are partly present in caveolae and are released from these structures upon activation. Phosphorylated EGFRs become ubiquitinated by the action of Cbl and are trapped by Eps15 in either the clathrin-coated vesicle (CCV) route or clathrin-independent route (Ci). Eps15 is associated to the ubiquitinated EGFR via its UIMs and to α-adaptin in the AP-2-complex in the CCV, and to a putative adaptor protein (?) at the clathrin independent pit. Eps15b is found at the early endosome (EE) in a tri-complex with STAM and Hrs bound to the clathrin-coated sheet. Inactive EGFRs are recycled back to the plasma membrane while active receptors are packed in multi vesicular vesicles (MVB) by the actions of the ESCRT-0, -I, -II, and -III complexes and degraded in lysosomes.

Since Eps15 is constitutively associated with the coated pit, the induced binding with the activated receptors would result in the recruitment of these receptors into the coated pit. This is in line with the observation that the EGFR is endocytosed via clathrin-coated vesicles However, accumulating evidence suggests that the EGFR can also be internalized via a less well defined, clathrin independent (Ci) pathway [68]. This is based on the fact that EGF internalization could not be completely abolished by inhibition of the CCV pathway, nor by over-expression of a dominant negative mutant of Eps15 or by silencing the clathrin heavy chain [68]. The dominant negative Eps15 variant lacks the EH domains, which result in a sequestration of α-adaptin and consequently in inhibition of CCV-mediated internalization [69]. Internalization of EGFR through the alternative pathway was linked to ubiquitination of the EGFR. Silencing of both Eps15 variants and epsin1 was found to block this alternative pathway demonstrating a role of these three proteins also in this clathrin-independent (Ci) internalization route (fig. 3) [68].

The function of Eps15 can best be described using a model of EGFR internalization. Activation of the EGFR results in cross-phosphorylation of tyrosine residues in the intracellular domain, causing the binding of SH2 containing proteins, such as Grb2 and the E3 ligase Cbl. As Grb2 also binds via its SH3 domain to a proline-rich motif in Cbl, a complex of EGFR/Grb2/Cbl is formed resulting in mono- and poly-ubiquitination of the EGFR [68]. This is followed by the UIM-dependent binding of Eps15 to the EGFR, resulting in the recruitment of EGFR to the coated pit (Fig. 3). In addition, Eps15 is binding via its EH domains to Epsin1, which may also bind to the poly-ubiquitinated EGFR via its UIM domains [70]. The lipid-binding EH and ENTH domains of Eps15 and Epsin-1 induce bending of the membrane. Further recruitment of these molecules to the pit consequently results in the further bending of the clathrin coated pit. In addition, also other binding partners of Eps15 are recruited to the pit and stimulate the formation of the coated vesicle further. Remarkably, after vesicle formation Eps15 seems to be absent from the vesicular structures [71]. Eps15 might equally well bind to the ubiquitinated EGFR in the Ci route, and stimulate, together with epsin1, the bending of the membrane (Fig. 3). Proteins mediating the Ci pathway, such as adaptor proteins, have not been identified yet, and are subject of further research. It should be noted, however, that the described Eps15 mediated internalization of the EGFR is not the only mechanism for EGFR internalization. For instance, EGFR dimerization has also been shown to be stimulate endocytosis [72]. Moreover, removal of the ubiquitination sites from the intracellular domain of the EGFR did not affect EGF internalization, indicating that several different internalization mechanism are acting synergistically in the internalization of EGFR [73].

Another suggested function for the Eps15 family members lays in the sorting process of EGFR that occurs at the membrane of the early endosome [74]. In this organelle, active, phosphorylated EGFRs are being separated from inactive receptors, a process named sorting. Four different protein complexes have been identified to be responsible for this, indicated as endosomal sorting complex required for transport or ESCRT complexes 0-, I, II, and III [75]. Ubiquitination of EGFR again plays an essential role in this process, which also involves the ubiquitin-binding proteins Hrs and STAM. Together, they form a complex with the ubiquitinated receptor at the membrane of the early endosome (ESCRT-0)[74]. The Eps15 isoform, Eps15b, binds preferentially to Hrs and is also localized to endosomes [29]. It was suggested that the Eps15b isoform functions specifically in complexes involved in sorting at the endosome, while Eps15 and Eps15R may function at the plasma membrane. The sorting process at the early endosome also involves the three other ESCRT complexes, which mediate the invagination of receptor containing vesicles into the endosome, resulting in the formation of multi-vesicular bodies (MVB) (Fig. 3) [76]. As these MVBs finally fuse with lysosomes, this pathway defines the degradation of the active, phosphorylated, and ubiquitinated EGFRs.

A very recent finding is that Eps15 also showed localization at the Golgi complex [11]. This is in agreement with observations that the appendage of the γ-adaptin subunit of the Golgi localized AP-1 complex binds to and colocalizes with Eps15 [45]. During further studies, a 14 amino acid motif was identified that is located immediately C-terminally from the AP-2 binding site in domain III of Eps15. The exit of secretory proteins was significantly reduced by silencing of γ-adaptin or overexpression of an Eps15 mutant lacking the particular AP-1 binding site [44]. This AP-1 binding site is also present in Eps15R and Eps15b, but data about a function of these Eps15 variants in secretion are so far lacking.

## Concluding remarks

Since its discovery in 1993, a function for Eps15 has been described in different processes, one related to a nuclear function and one related to intracellular trafficking. In trafficking, Eps15 acts as an ubiquitin- or Ubl-binding adaptor protein, a function mediated by the two UIMs in the C-terminal part of the molecule. Other domains in Eps15 are involved in the recruitment of different components that are critical for trafficking, including α- and γ-adaptin, Epsin1, synaptojanin etc. In these processes, Eps15 functions as a scaffolding protein that recruits and concentrates those proteins and lipids that are required for the process. Post-translational modifications as phosphorylation and mono-ubiquitination might regulate Eps15 functioning, but molecular details of these processes are still unknown. Similarly, details about the function of Eps15b in the ESCRT-0 complex is not clear. Imaging of the internalization processes at high resolution may help to understand the molecular details of these processes in the future. The trafficking role of the Eps15 molecules includes clearly two aspects: they are bound to membranes and the morphology of these membranes are manipulated. This has also been described for EHD1, an EH domain containing protein that induces tubulation of the membrane [39]. This suggest a general role for Eps15 and its family members in controlling membrane morphology during the process of intracellular trafficking.

## Competing interests

The author declares that he has no competing interests.
